# Haematopoietic stem cells: past, present and future

**DOI:** 10.1038/cddiscovery.2017.2

**Published:** 2017-02-06

**Authors:** Ashley P Ng, Warren S Alexander

**Affiliations:** 1 The Walter and Eliza Hall Institute of Medical Research, 1G Royal Parade, Parkville, VIC, Australia; 2 Department of Medical Biology, The University of Melbourne, Parkville, VIC, Australia

## Abstract

The discovery and characterisation of haematopoietic stem cells has required decades of research. The identification of adult bone marrow as a source of haematopoietic cells capable of protecting an organism from otherwise lethal irradiation led to the intense search for their identity and characteristics. Using functional assays along with evolving techniques for isolation of haematopoietic cells, haematopoietic stem cell populations were able to be enriched and their characteristics analysed. The key haematopoietic stem cell characteristics of pluripotentiality and the ability for self-renewal have emerged as characteristics of several haematopoietic stem cell populations, including those that have recently challenged the conventional concepts of the haematopoietic hierarchy. Human allogeneic stem cell therapy relies on these functional characteristics of haematopoietic stem cells that can be isolated from peripheral blood, bone marrow or cord blood, with the additional requirement that immunological barriers need to be overcome to allow sustained engraftment while minimising risk of graft-versus-host disease developing in the recipient of transplanted stem cells. Current and future research will continue to focus on the identification of haematopoietic stem cell regulators and methods for *in vitro* and *in vivo* stem cell manipulation, including genome editing, to expand the scope, potential and safety of therapy using haematopoietic stem cells.

## key-points

- Haematopoietic stem cells are rare cells with characteristics of pluripotency and self-renewal that are capable of generating an entire haematopoeitic system.
- Haematopoietic stem cells have been identified at defined stages of embryonic development and several subsets have been characterised in adult haematopoiesis.
- The relative contribution of haematopoietic stem cells to steady-state and stress haematopoiesis remains controversial.
- Allogeneic haematopoietic stem cell transplantation therapy in human requires significant immunological barriers to be overcome.
- Current and future research aims to identify key stem cell regulators and methods for *in vitro* and *in vivo* stem cell manipulation including genome editing.

Haematopoietic stem cells (HSC) are the architects of definitive haematopoiesis, that is, blood cell production that occurs continuously during the life of an organism. Each HSC is programmed to allow efficient production of the cellular blood components with a manifest purpose that has been shaped by evolution: from red cells that allow efficient carriage of oxygen, megakaryocytes and their platelet progeny that interact with blood vessels and soluble coagulation factors to regulate clotting, to the cells of the innate and acquired immune system that act against microbial attack. HSCs are defined by their pluripotentiality, the capacity for a single HSC to generate any and all of the diverse mature functional haematopoietic cell types. Key genes and select genetic programmes are invoked for the maintenance, or self-renewal of HSCs and for the formation of the specific haematopoietic lineages.^1–5^

**Figure 1:**
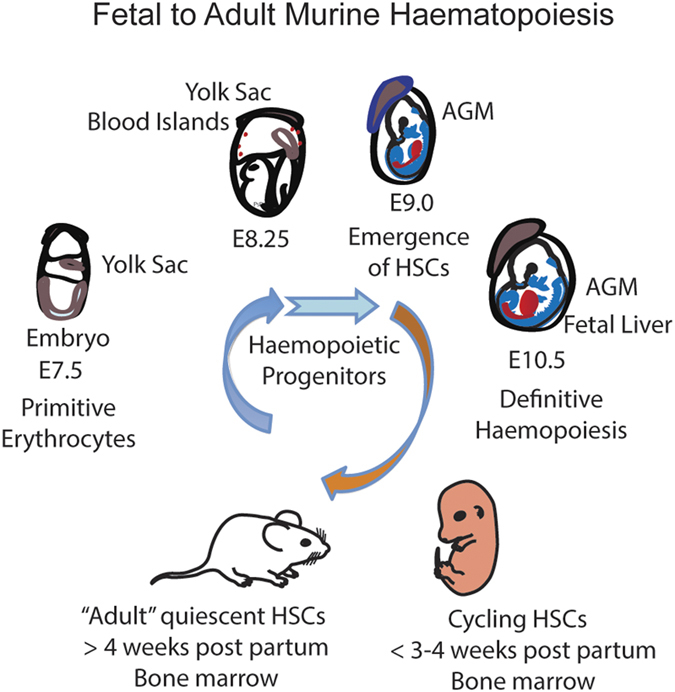
The journey from fetal to adult haematopoiesis adapted from Dzierzak and Speck.^8^ AGM, aorto-gonado-mesonephros; PsP, para-aortic-splanchnopleura. See references in main text.

Definitive haematopoiesis in the embryo begins with the emergence of the first identifiable HSC in the aorto-gonado-mesonephros region.^6,7^ Thereafter, haematopoiesis shifts to the fetal liver, and subsequently to the bone marrow, where HSCs will reside for the life of the mammalian organism^8,9^ (Figure 1).

## Identification of HSCs

It became apparent, initially through work that sought to characterise radiation sensitivity, that donor adult bone marrow transplanted into syngeneic irradiated murine recipients was capable of protecting the recipients from lethal irradiation by regenerating (reconstituting) the irradiation-ablated haematopoietic system. This research was crucial in the development of the concept of HSC as cells in the bone marrow capable of generating the complete blood cell system, although at this time the specific cell had yet to be isolated and characterised.

In these early experiments, donor-derived clonogenic colonies of multiple haematopoietic lineages were able to be macroscopically identified in the spleen of transplanted recipients.^10,11^ These spleen colony-forming units, although not definitive HSCs, were nevertheless useful in allowing characterisation of progenitor cells responsible for haematopoietic reconstitution. Specific progenitor cells appeared to possess the ability to form multiple haematopoietic lineages from within the one colony (multipotency), while others appeared able to form daughter cells that retained the characteristics of the original parental cell (self-renewal).^12^ These two important characteristics ultimately came to be recognised as definitive characteristics of HSC.

The identification and characterisation of HSCs ultimately required strategies to separate these rare bone marrow cells from more numerous cellular components. Functional competitive repopulating unit assays estimated the frequency of these rare cells as one in 10 000 cells in bone marrow.^13^ Like the proverbial search for the needle in a haystack, HSCs were eventually isolated with increasing purity based on physical properties, such as Hoescht 33342 supravital dye exclusion,^14^ resistance to 5-fluorouracil^15^ or *γ*-irradiation.^16^ Ultimately, however, it was the application of flow cytometry and the use of specific cell surface antigen markers^17^ that led to the ability to prospectively identify cell populations able to reconstitute multiple lineages upon transplantation, and capable of self-renewal as judged by serial transplantation assays. These cell populations, enriched for HSCs, were notable for their lack of mature lineage antigen expression, and expression of antigens such as cKit, the cellular receptor for the cytokine stem cell factor.^18,19^

**Figure 2:**
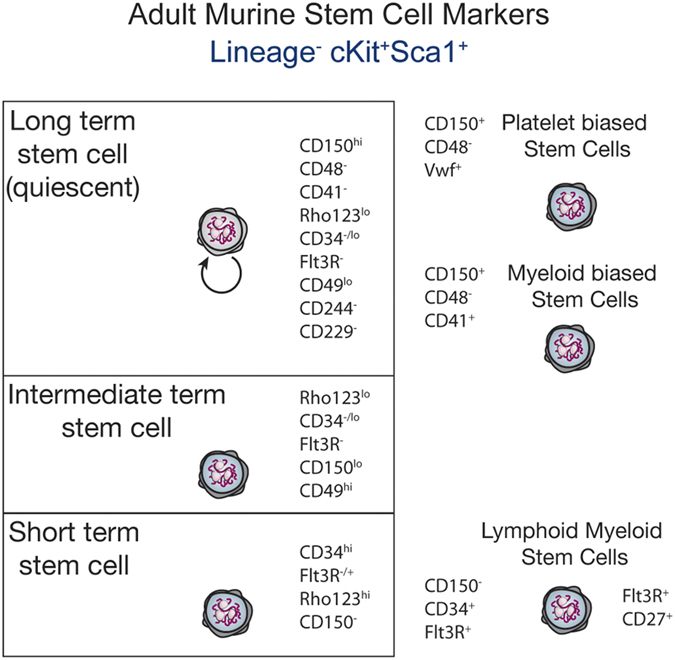
Immunophenotypic markers of adult murine HSCs and ‘lineage-restricted’ HSC populations. See references in main text.

HSCs were found to possess unique properties that set them apart from other blood-forming progenitor cells. In addition to the properties of pluripotency and self-renewal, adult long-term HSCs were found to reside in a specific niche environment in the bone marrow, which was closely associated with endosteum,^20^ and where they exist in conditions of relative hypoxia.^21^ Here, HSC exists predominantly in a non-replicative and quiescent state,^22^ in which signalling by the cytokine thrombopoietin^23,24^ and the presence of megakaryocytes are recognised to have an important role.^25–27^ In contexts that place the haematopoietic system under stress, such as chronic infection, these quiescent stem cells are recruited into cell cycle, for example, via interferon signalling, which is associated with a numerical increase in downstream progenitor cells.^28^ Evidence increasingly suggests that lineage specification can occur very early in the haematopoietic hierarchy in immunophenotypically defined ‘stem cell’ populations^29–32^ (Figure 2), supporting findings that self-renewing lineage-restricted progenitors may emerge directly from HSC.^33^ A significant degree of complexity may therefore exist in the pathways via which mature haematopoietic lineages develop from HSC and progenitor populations. More direct pathways from HSC to specific mature cells may coexist alongside traditional models of progenitor population hierarchies. Recent evidence from clonal dynamic studies tracing the origin of blood cells over time has suggested that steady-state blood cell maintenance does not incessantly call upon the quiescent HSCs to enter into cell cycle, but rather, successive recruitment of long-lived progenitor populations appears to primarily maintain blood cells at steady state.^34^ However, other studies have yielded apparently conflicting results on the relative contribution of HSCs to steady state and stress haematopoiesis,^35,36^ a current controversy that remains to be resolved.

In the setting of bone marrow transplantation, which is dependent on HSCs to sustainably reconstitute haematopoiesis, further refinement of cell surface markers have also identified specific subsets of HSCs with more limited capacity for self-renewal, yet important for maintaining haematopoiesis in the short and intermediate terms after transplantation.^37,38^ Stem cells capable of true long-term reconstitution with durable self-renewal potential do appear to be a very rare but essential cell population required for long-term haematopoietic engraftment^39–41^ (Figure 2).

## Bone marrow transplantation as therapy

**Figure 3:**
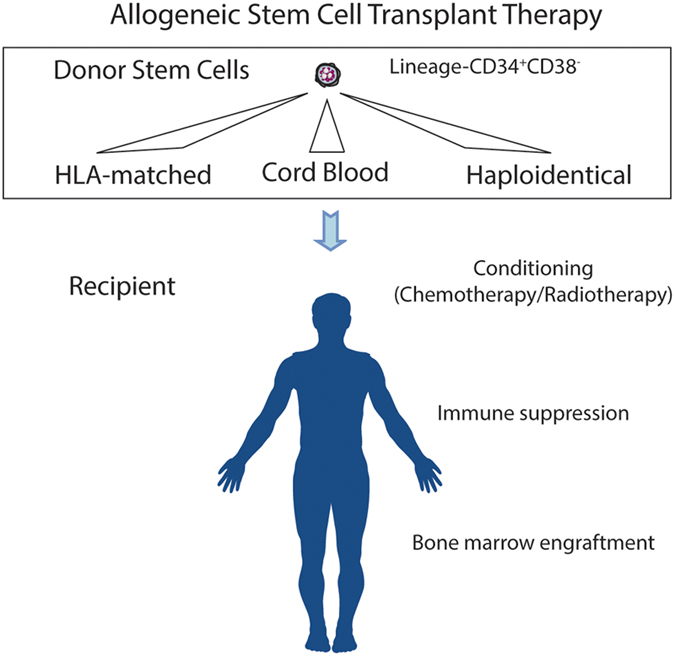
Human HSC transplantation therapy. HLA-matched adult, cord blood or haploidentical adult donor stem and progenitor cells are transplanted intravenously into a recipient following conditioning therapy to permit engraftment of donor marrow into the recipient. Immune suppression is administered to prevent acute graft-versus-host disease.

Although murine haematopoiesis reflects human haematopoiesis in many ways, the immunophenotypic markers of human HSCs (Lineage^−^CD34^+^CD38^−^) differ from functionally similar murine counterparts.^42^ Unlike inbred genetically and immunologically identical mouse strains, successful allogeneic transplantation therapy in humans requires significant immunological barriers to be overcome. The discovery of the HLA system of MHC class I and II receptors, which engage T-cell antigen receptors, allows histocompatible matching of donors and recipients. This is supplemented with the use of immunosuppression during and after the transplantation of allogeneic stem cells from volunteer-related and -unrelated donors. Advancement of stem cell transplantation therapy has focussed on research to broaden the availability of donors to patients. Use of cord blood units as a source of stem cells^43,44^ and recently developed conditioning and immunosuppressive regimens have allowed haploidentical transplantation^45^ to become a therapeutic reality while limiting the immunological consequence of graft-versus-host disease. These approaches are increasingly making the option of allogeneic transplantation available to patients who otherwise do not have a matched-related or volunteer-unrelated donor source of stem cells (Figure 3).

## The future

Research defining the nature and regulation of HSCs has allowed regulators of blood cell production to be manipulated in ways that have revolutionised treatment of blood disorders and the use of stem cell transplants. Novel outcomes from ongoing stem cell research continue to refine this understanding and provides the avenue to continual treatment improvements.

**Figure 4:**
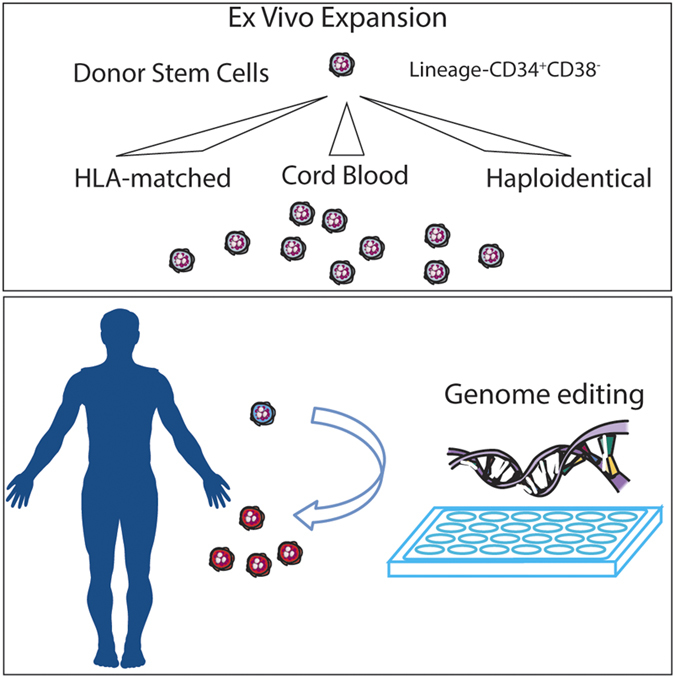
Future directions and applications of HSC research. (Top panel) *Ex vivo* stem cell expansion. (Bottom panel) Genome editing of HSCs.

Important challenges remain. These include: developing robust methods to maintain HSCs *in vitro* both to enhance research and to expand cell numbers for therapy; developing a deeper understanding of the HSC niche and intrinsic and extrinsic HSC regulators; and to develop safely the future capacity to ‘reprogramme’ cells to HSCs, correct genetically defective HSCs that would allow transplantation of ‘corrected’ syngeneic patient cells or transplant reprogrammed haematopoietic cells for directed therapy against specific patient diseases (Figure 4).

## References

[bib1] Ivanova NB, Dimos JT, Schaniel C, Hackney JA, Moore KA, Lemischka IR. A stem cell molecular signature. Science 2002; 298: 601–604. 12228721 10.1126/science.1073823

[bib2] Mercer EM, Lin YC, Murre C. Factors and networks that underpin early hematopoiesis. Semin Immunol 2011; 23: 317–325. 21930392 10.1016/j.smim.2011.08.004 PMC3217090

[bib3] Novershtern N, Subramanian A, Lawton LN, Mak RH, Haining WN, McConkey ME et al. Densely interconnected transcriptional circuits control cell states in human hematopoiesis. Cell 2011; 144: 296–309. 21241896 10.1016/j.cell.2011.01.004 PMC3049864

[bib4] Moignard V, Macaulay IC, Swiers G, Buettner F, Schütte J, Calero-Nieto FJ et al. Characterization of transcriptional networks in blood stem and progenitor cells using high-throughput single-cell gene expression analysis. Nat Cell Biol 2013; 15: 363–372. 23524953 10.1038/ncb2709 PMC3796878

[bib5] Riddell J, Gazit R, Garrison BS, Guo G, Saadatpour A, Mandal PK et al. Reprogramming committed murine blood cells to induced hematopoietic stem cells with defined factors. Cell 2014; 157: 549–564. 24766805 10.1016/j.cell.2014.04.006 PMC4060823

[bib6] Ivanovs A, Rybtsov S, Welch L, Anderson RA, Turner ML, Medvinsky A. Highly potent human hematopoietic stem cells first emerge in the intraembryonic aorta-gonad-mesonephros region. J Exp Med 2011; 208: 2417–2427. 22042975 10.1084/jem.20111688 PMC3256972

[bib7] Ivanovs A, Rybtsov S, Anderson RA, Turner ML, Medvinsky A. Identification of the niche and phenotype of the first human hematopoietic stem cells. Stem Cell Rep 2014; 2: 449–456. 10.1016/j.stemcr.2014.02.004 PMC3986508 24749070

[bib8] Dzierzak E, Speck NA. Of lineage and legacy: the development of mammalian hematopoietic stem cells. Nat Immunol 2008; 9: 129–136. 18204427 10.1038/ni1560 PMC2696344

[bib9] Medvinsky A, Rybtsov S, Taoudi S. Embryonic origin of the adult hematopoietic system: advances and questions. Development 2011; 138: 1017–1031. 21343360 10.1242/dev.040998

[bib10] Till JE, McCulloch EA. A direct measurement of the radiation sensitivity of normal mouse bone marrow cells. Radiat Res 1961; 14: 213–222. 13776896

[bib11] Becker AJ, McCulloch EA, Till JE. Cytological demonstration of the clonal nature of spleen colonies derived from transplanted mouse marrow cells. Nature 1963; 197: 452–454. 13970094 10.1038/197452a0

[bib12] Siminovitch L, McCulloch EA, Till JE. The distribution of colony-forming cells among spleen colonies. J Cell Physiol 1963; 62: 327–336. 10.1002/jcp.1030620313 14086156

[bib13] Szilvassy SJ, Humphries RK, Lansdorp PM, Eaves AC, Eaves CJ. Quantitative assay for totipotent reconstituting hematopoietic stem cells by a competitive repopulation strategy. Proc Natl Acad Sci USA 1990; 87: 8736–8740. 2247442 10.1073/pnas.87.22.8736 PMC55034

[bib14] Goodell MA, Brose K, Paradis G, Conner AS, Mulligan RC. Isolation and functional properties of murine hematopoietic stem cells that are replicating *in vivo*. J Exp Med 1996; 183: 1797–1806. 8666936 10.1084/jem.183.4.1797 PMC2192511

[bib15] Hodgson GS, Bradley TR. Properties of haematopoietic stem cells surviving 5-fluorouracil treatment: evidence for a pre-CFU-S cell? Nature 1979; 281: 381–382. 481601 10.1038/281381a0

[bib16] Ploemacher RE, van Os R, van Beurden CA, Down JD. Murine haemopoietic stem cells with long-term engraftment and marrow repopulating ability are more resistant to gamma-radiation than are spleen colony forming cells. Int J Radiat Biol 1992; 61: 489–499. 1349331 10.1080/09553009214551251

[bib17] Spangrude GJ, Heimfeld S, Weissman IL. Purification and characterization of mouse hematopoietic stem cells. Science 1988; 241: 58–62. 2898810 10.1126/science.2898810

[bib18] Okada S, Nakauchi H, Nagayoshi K, Nishikawa S, Nishikawa S, Miura Y et al. Enrichment and characterization of murine hematopoietic stem cells that express c-kit molecule. Blood 1991; 78: 1706–1712. 1717068

[bib19] Morrison SJ, Weissman IL. The long-term repopulating subset of hematopoietic stem cells is deterministic and isolatable by phenotype. Immunity 1994; 1: 661–673. 7541305 10.1016/1074-7613(94)90037-x

[bib20] Morrison SJ, Scadden DT. The bone marrow niche for haematopoietic stem cells. Nature 2014; 505: 327–334. 24429631 10.1038/nature12984 PMC4514480

[bib21] Nombela-Arrieta C, Pivarnik G, Winkel B, Canty KJ, Harley B, Mahoney JE et al. Quantitative imaging of haematopoietic stem and progenitor cell localization and hypoxic status in the bone marrow microenvironment. Nat Cell Biol 2013; 15: 533–543. 23624405 10.1038/ncb2730 PMC4156024

[bib22] Wilson A, Laurenti E, Oser G, van der Wath RC, Blanco-Bose W, Jaworski M et al. Hematopoietic stem cells reversibly switch from dormancy to self-renewal during homeostasis and repair. Cell 2008; 135: 1118–1129. 19062086 10.1016/j.cell.2008.10.048

[bib23] Qian H, Buza-Vidas N, Hyland CD, Jensen CT, Antonchuk J, Mansson R et al. Critical role of thrombopoietin in maintaining adult quiescent hematopoietic stem cells. Cell Stem Cell 2007; 1: 671–684. 18371408 10.1016/j.stem.2007.10.008

[bib24] Yoshihara H, Arai F, Hosokawa K, Hagiwara T, Takubo K, Nakamura Y et al. Thrombopoietin/MPL signaling regulates hematopoietic stem cell quiescence and interaction with the osteoblastic niche. Cell Stem Cell 2007; 1: 685–697. 18371409 10.1016/j.stem.2007.10.020

[bib25] Zhao M, Perry JM, Marshall H, Venkatraman A, Qian P, He XC et al. Megakaryocytes maintain homeostatic quiescence and promote post-injury regeneration of hematopoietic stem cells. Nat Med 2014; 20: 1321–1326. 25326798 10.1038/nm.3706

[bib26] Bruns I, Lucas D, Pinho S, Ahmed J, Lambert MP, Kunisaki Y et al. Megakaryocytes regulate hematopoietic stem cell quiescence through CXCL4 secretion. Nat Med 2014; 20: 1315–1320. 25326802 10.1038/nm.3707 PMC4258871

[bib27] Nakamura-Ishizu A, Takubo K, Kobayashi H, Suzuki-Inoue K, Suda T. CLEC-2 in megakaryocytes is critical for maintenance of hematopoietic stem cells in the bone marrow. J Exp Med 2015; 212: 2133–2146. 26552707 10.1084/jem.20150057 PMC4647260

[bib28] Baldridge MT, King KY, Boles NC, Weksberg DC, Goodell MA. Quiescent haematopoietic stem cells are activated by IFN-gamma in response to chronic infection. Nature 2010; 465: 793–797. 20535209 10.1038/nature09135 PMC2935898

[bib29] Adolfsson J, Mansson R, Buza-Vidas N, Hultquist A, Liuba K, Jensen CT et al. Identification of Flt3+ lympho-myeloid stem cells lacking erythro-megakaryocytic potential a revised road map for adult blood lineage commitment. Cell 2005; 121: 295–306. 15851035 10.1016/j.cell.2005.02.013

[bib30] Serwold T, Ehrlich LI, Weissman IL. Reductive isolation from bone marrow and blood implicates common lymphoid progenitors as the major source of thymopoiesis. Blood 2009; 113: 807–815. 18927436 10.1182/blood-2008-08-173682 PMC4123410

[bib31] Sanjuan-Pla A, Macaulay IC, Jensen CT, Woll PS, Luis TC, Mead A et al. Platelet-biased stem cells reside at the apex of the haematopoietic stem-cell hierarchy. Nature 2013; 502: 232–236. 23934107 10.1038/nature12495

[bib32] Pietras EM, Reynaud D, Kang YA, Carlin D, Calero-Nieto FJ, Leavitt AD et al. Functionally distinct subsets of lineage-biased multipotent progenitors control blood production in normal and regenerative conditions. Cell Stem Cell 2015; 17: 35–46. 26095048 10.1016/j.stem.2015.05.003 PMC4542150

[bib33] Yamamoto R, Morita Y, Ooehara J, Hamanaka S, Onodera M, Rudolph KL et al. Clonal analysis unveils self-renewing lineage-restricted progenitors generated directly from hematopoietic stem cells. Cell 2013; 154: 1112–1126. 23993099 10.1016/j.cell.2013.08.007

[bib34] Sun J, Ramos A, Chapman B, Johnnidis JB, Le L, Ho YJ et al. Clonal dynamics of native haematopoiesis. Nature 2014; 514: 322–327. 25296256 10.1038/nature13824 PMC4408613

[bib35] Schoedel KB, Morcos MN, Zerjatke T, Roeder I, Grinenko T, Voehringer D et al. The bulk of the hematopoietic stem cell population is dispensable for murine steady-state and stress hematopoiesis. Blood 2016; 128: 2285–2296. 10.1182/blood-2016-03-706010 27357698

[bib36] Sawai CM, Babovic S, Upadhaya S, Knapp DJ, Lavin Y, Lau CM et al. Hematopoietic stem cells are the major source of multilineage hematopoiesis in adult animals. Immunity 2016; 45: 597–609. 27590115 10.1016/j.immuni.2016.08.007 PMC5054720

[bib37] Yang L, Bryder D, Adolfsson J, Nygren J, Mansson R, Sigvardsson M et al. Identification of Lin(−)Sca1(+)kit(+)CD34(+)Flt3− short-term hematopoietic stem cells capable of rapidly reconstituting and rescuing myeloablated transplant recipients. Blood 2005; 105: 2717–2723. 15572596 10.1182/blood-2004-06-2159

[bib38] Benveniste P, Frelin C, Janmohamed S, Barbara M, Herrington R, Hyam D et al. Intermediate-term hematopoietic stem cells with extended but time-limited reconstitution potential. Cell Stem Cell 2010; 6: 48–58. 20074534 10.1016/j.stem.2009.11.014

[bib39] Morita Y, Ema H, Nakauchi H. Heterogeneity and hierarchy within the most primitive hematopoietic stem cell compartment. J Exp Med 2010; 207: 1173–1182. 20421392 10.1084/jem.20091318 PMC2882827

[bib40] Oguro H, Ding L, Morrison SJ. SLAM family markers resolve functionally distinct subpopulations of hematopoietic stem cells and multipotent progenitors. Cell Stem Cell 2013; 13: 102–116. 23827712 10.1016/j.stem.2013.05.014 PMC3736853

[bib41] Wilson NK, Kent DG, Buettner F, Shehata M, Macaulay IC, Calero-Nieto FJ et al. Combined single-cell functional and gene expression analysis resolves heterogeneity within stem cell populations. Cell Stem Cell 2015; 16: 712–724. 26004780 10.1016/j.stem.2015.04.004 PMC4460190

[bib42] Larochelle A, Savona M, Wiggins M, Anderson S, Ichwan B, Keyvanfar K et al. Human and rhesus macaque hematopoietic stem cells cannot be purified based only on SLAM family markers. Blood 2011; 117: 1550–1554. 21163926 10.1182/blood-2009-03-212803 PMC3318774

[bib43] Delaney C, Gutman JA, Appelbaum FR. Cord blood transplantation for haematological malignancies: conditioning regimens, double cord transplant and infectious complications. Br J Haematol 2009; 147: 207–216. 19796270 10.1111/j.1365-2141.2009.07782.x

[bib44] Munoz J, Shah N, Rezvani K, Hosing C, Bollard CM, Oran B et al. Concise review: umbilical cord blood transplantation: past, present, and future. Stem Cells Transl Med 2014; 3: 1435–1443. 25378655 10.5966/sctm.2014-0151 PMC4250219

[bib45] Piemontese S, Ciceri F, Labopin M, Bacigalupo A, Huang H, Santarone S et al. A survey on unmanipulated haploidentical hematopoietic stem cell transplantation in adults with acute leukemia. Leukemia 2015; 29: 1069–1075. 25434302 10.1038/leu.2014.336

